# *In Vitro* Role of Pumpkin Parts as Pharma-Foods: Antihyperglycemic and Antihyperlipidemic Activities of Pumpkin Peel, Flesh, and Seed Powders, in Alloxan-Induced Diabetic Rats

**DOI:** 10.1155/2022/4804408

**Published:** 2022-08-02

**Authors:** Ashiq Hussain, Tusneem Kausar, Muhammad Abdullah Jamil, Saima Noreen, Khansa Iftikhar, Ayesha Rafique, Muhammad Azhar Iqbal, Muhammad Abid Majeed, Muhammad Yousaf Quddoos, Jawed Aslam, Atif Ali

**Affiliations:** ^1^Institute of Food Science and Nutrition, University of Sargodha, Sargodha, Pakistan; ^2^Institute of Food and Nutritional Sciences, Pir Mehr Ali Shah Arid Agriculture University, Rawalpindi, Pakistan

## Abstract

Pumpkin is a well-known vegetable, among the members of *Cucurbitaceae* family, due to its importance as pharma food. Keeping in view the antidiabetic and plasma lipids lowering potential of pumpkin, the present study was conducted to investigate that, which part of pumpkin (peel, flesh, and seeds), possess more bioactive compounds, exhibiting antihyperglycemic and antihyperlipidemic potential. Albino rats with 190-210 g body weight were divided into 11 groups. Five rats were included in each group; group A was negative control, group B was positive control, and groups C to K were diabetic rats fed with pumpkin peel, flesh, and seed powders. Diabetes was induced in rats with the help of alloxan monohydrate. During 28 days of experimental period, blood glucose level of different rat's groups was checked with the help of glucometer, at every 7 days interval and at the end of 28 days study, plasma lipids were checked with the help of commercial kits. A significant decrease in blood glucose level (128.33 ± 1.67 mg/dl), TC (88.43 ± 0.66 mg/dl), TG (69.79 ± 0.49 mg/dl), and LDL-C (21.45 ± 0.08 mg/dl) was recorded in rat groups fed with 15 g pumpkin seed powder, at the end of study. After pumpkin seeds, second significant antihyperglycemic and antihyperlipidemic effect was recorded in rat's groups fed with 15 g pumpkin peel powder. Pumpkin flesh powder effect in lowering blood glucose level and plasma lipids was less significant as compared to seeds and peel powder. As the dose of the pumpkin powders was increased from 5 to 10 and then 15 g, the blood glucose-lowering and plasma lipid-lowering effect became more significant. Similarly, as the experimental duration was expanded from first week to 28 days, this antihyperglycemic and antihyperlipidemic effect became more significant. These results were sufficient to conclude that pumpkin has high potential to be used in human diet to cope with noncommunicable diseases like diabetes and hypercholesterolemia.

## 1. Introduction

Fruit and vegetable processing industries produce huge amount of waste materials every year creating environmental pollution. These by-products are an excellent source of bioactive compounds including carbohydrates, lignin, protein, fat, and phenolics, which have potential to play role as medicinal components [1]. Expectations of consumers from food industry are food products, which play both functional and medicinal role in the body. Pumpkin has strong potential to overcome food insecurity and generate income [2]. Extracts from pumpkin fractions were evaluated for total phenolic, flavonoid, carotenoid, and mineral contents, and results revealed excellent phytochemical profiles of peel, flesh, and seeds of pumpkin [3]. Global pumpkin, squash, and gourd production in 2019 was estimated above 23 million tons comprising an area of 1.54 million hectare. In Pakistan, this production was estimated 2.7 lac tons cultivated on 26515-hectare area [4].

Pumpkin is a gourd-like fruit native of tropical and subtropical regions, well known for its excellent nutritional profile. Most common natural phytochemicals present in sufficient quantities in pumpkin are carotenoids, phenolics, vitamins, minerals, polysaccharides, pectins, fibers, tocopherols, phytosterols, essential oils, proteins, peptides, and amino acids [5]. Pumpkin belongs to *Cucurbitaceae* family, and members of this family participate in promoting human and animal health due to biological activities of their phytochemicals [6]. Pumpkin has potential to act as functional food as an important source of carotenoids, vitamins, and minerals [7]. Different varieties of pumpkin possess strong antioxidant potential [8]. Phenolic compounds present in pumpkin seeds exert a number of health benefits mainly due to their antioxidant potential [9]. Seeds of pumpkins are found to be an excellent source of vitamins, minerals, phenolic compounds, antioxidants, carotenoids, proteins, and essential oils, responsible for positive health impacts in humans [10]. Pumpkin seed kernel flour has high protein, oil, and oleic and linoleic acids [11]. Extracts from different parts of the pumpkin contain biologically active compounds [12]. Pumpkin seed oil press cake flour could be used as functional potential substitute of wheat flour [13].

Plant-derived polysaccharides from pumpkin reduce the oxidative stress level in cells and tissues and could decrease the diabetes symptoms in animals as well as humans [14]. Reports of pumpkin of lowering blood glucose level in alloxan-induced diabetic rabbits, temporarily hypoglycaemic rabbits and type 2 diabetic patients, are present in studies [15, 16]. Xia and Wang [17] conducted research on hypoglycaemic effect of pumpkin by using streptozocin-diabetic rats. Development of nutraceuticals and value-added food products by utilization of pumpkin-based components is very important, due to its antidiabetic and anticarcinogenic activities [18]. Pumpkin plays an important role in human health by acting as medicinal food because it has potential of antidiabetic, antioxidant, antimicrobial, anticarcinogenic, and anti-inflammatory agent [19]. From pumpkin fruit pulp, a variety of polysaccharides have been recovered having hypoglycaemic potential [20].

Diabetes mellitus is a chronic metabolic disorder which is due to multifarious aetiologies [21]. Among the causes of diabetes mellitus, one is disturbance of metabolism of carbohydrates, proteins, and fats [22]. Usage of dietary plants and herbal extracts instead of Western medicine in preventing and treating diabetes mellitus has gained attention worldwide [23]. Herbal extracts are used to treat diabetes mellitus in Mexico. These natural extracts are supposed to have significant beneficial effect at early stages of diabetes mellitus [16]. More than 200 plant species including pumpkin have been confirmed to show hypoglycemic characteristics by Chinese herbal drugs [24]. Pumpkins are traditionally used as medicine for diabetes in numerous other countries like India, America, Brazil, and Argentina [25].

Many dietary sources have now received significant attention throughout the world because of the potential benefits they possess in regard to various disorders like diabetes. Among these sources, pumpkin seeds have been utilized as new components of traditional health foods in Tunisia and some other countries of North Africa [26]. Active-hypoglycemic agents that can be obtained from pumpkin are seed oils, pectin, hypoglycemic proteins, and nonpectin polysaccharides .

Pumpkin seed oil having high oxidative stability is rich source of plant sterols and unsaturated fatty acids, which possess potential of cardiovascular benefits [27]. Pumpkin seed oil is a rich natural source of antioxidant vitamins, tocopherols, and phytosterols and recommended to be used in human diet for health as it mitigates hypercholesterolemia and hypercholesterolemia [28]. Pumpkin seeds are rich source of essential nutrients posing positive impacts on health [29]. Oils extracted from raw pumpkin seeds significantly reduced serum triglycerides, total cholesterols, LDL cholesterols, uric acid, creatinine, serum transaminases, and urea, whereas serum HDL cholesterols were significantly increased [30]. Pumpkin peel and seed mixtures when extracted presented high concentrations of oleic, linoleic, and linolenic acids [31]. Pumpkin peel, flesh, and seed powder extracts exhibited strong antioxidant and antimicrobial activities [32]. Pumpkin peel, flesh, and seeds were separated, dried into powders, and utilized to develop good quality bakery product [33]. Research conducted by Chen et al. [34] provides useful information for understanding the inhibitory effects of polysaccharides from pumpkin on LDL oxidation. Proper pretreatments of pumpkin fruit pulp before drying retained optimum medicinal properties of this fruit by retaining maximum bioactives [35]. [36] conducted research on alloxan-induced diabetic rats, and the results revealed that pumpkin flour had the hypoglycemic and hypolipidemic effect. After comprehensive study of publications made by scientists and researchers on blood glucose- and serum lipid-lowering effects of pumpkin, the aims of the present research work were to investigate that among peel, flesh, and seeds, which part of pumpkin, have more potential as antihyperglycemic and antihyperlipidemic activities, in alloxan-induced diabetic rats.

## 2. Materials and Methods

### 2.1. Procurement of Materials, Chemicals, and Animals

Ripe pumpkins (*n* = 50) with an average weight of 5 ± 0.5 kg were purchased from the local market of District Sargodha, Pakistan. Albino rats with 190-210 g body weight of either sex were purchased from the National Institute of Health Sciences, Islamabad, Pakistan. All chemicals used in this research work were of reagent grade and purchased from the Sigma-Aldrich Company, Germany. Alloxan monohydrate, syringes for injections, feed for rats, glucometer to check blood glucose level, and commercial kits to analyze plasma lipids were purchased from scientific stores and local pharmacies of District Sargodha, Pakistan.

### 2.2. Preparation of Pumpkin Peel, Flesh, and Seed Powders

Pumpkins were washed manually, peeled, and separated into three fractions, i.e., peel, flesh, and seeds. Slicing of flesh portion into 2 × 3 inch pieces was done with the help of knife. After slicing, blanching at 94°C for 2 minutes was performed to inactivate enzymes. Powder of each fraction was prepared by conventional hot air-drying method, at 60°C for 24 hours in hot air oven (BIOBASE HAS-T105 China). Grinding of dried parts was done with common spice grinder (NIMA NM-8300 Japan), to obtain fine quality powder as described by Pongjanta et al. (2006), with some modifications. Final powders were packed in polyethylene bags and stored at ambient conditions.

### 2.3. Biological Study of Pumpkin Peel, Flesh, and Seed Powders

#### 2.3.1. Experimental Model

Experimental model was designed by following the procedure adopted by Bukhari et al. [37]. Albino rats with 190-210 g body weight of either sex were maintained under standard laboratory conditions in propylene cages at 25 ± 3°C, RH 50 ± 10% under 12 hours light/dark cycle. Before the onset of experimental procedure, the normal diet was fed to the rats, kept under observation.

#### 2.3.2. Experimental Design

**Figure 1:**
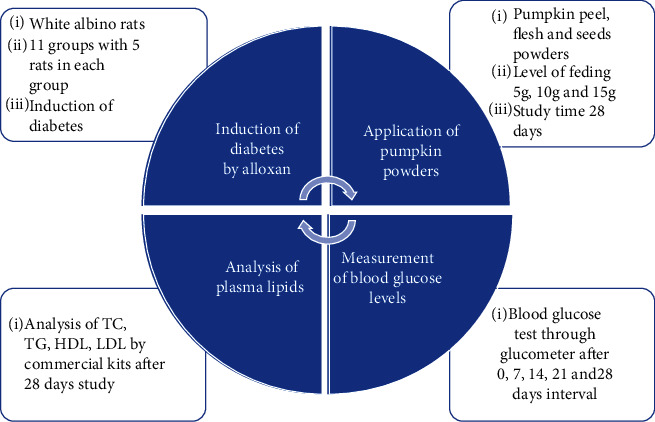
An overview of research work plan.

The experimental study was conducted for a period of 28 days. Experimental design included 11 groups comprised of 5 rats in each group: group A was negative control (normal rats with normal diet), group B was positive control (diabetic rats with normal diet), and groups C-K were nine groups of diabetic rats fed with 5, 10, and 15 g of each three types of pumpkin peel, flesh, and seed powders. Treatment's plan is given below in Table 1, and Figure 1 presents an overview of research work plan.

#### 2.3.3. Induction of Diabetes

After setting of experimental design, diabetes was induced in selected rat's groups, other than negative control, through intraperitoneal injection of freshly prepared alloxan monohydrate solution, in normal saline at dose of 120 mg kg^−1^ body weight. Time of one week was provided to the alloxan-injected rats to stabilize the diabetes under controlled conditions [38]. Rats having moderate diabetes that revealed glycosuria and hyperglycemia (i.e., blood glucose concentration 200–300 mg dl^−1^) were taken for the experimental tests.

#### 2.3.4. Blood Glucose Test

On days 7, 14, and 21 of the experiment and terminally on day 28, blood glucose level of all rat groups under study was checked by using glucometer (My-G025m China) as elaborated by Bukhari et al. [37], and all tests were performed in triplicate. An overview of research has been presented in Figure 1.

#### 2.3.5. Analysis of Plasma Lipids

Extraction of plasma lipids was done with the help of chloroform/methanol mixture (2 : 1, *v*/*v*) by following the method described by Yassin et al. [39]. Total lipid contents in plasma extracts were gravimetrically determined through solvent evaporation with the help of rotary evaporator (BIOLAND RE-5000A China). Parameters of plasma lipids like total cholesterol (TC), high-density lipoprotein cholesterol (HDL-C), and triacylglycerol (TG) levels were calculated through enzymatic method, with the help of commercial kits (TR0100, MAK045, MAK175 Sigma-Aldrich, Germany). Low-density lipoprotein cholesterol (LDL-C) was quantified by the Friedewald equation (Friedewald et al., 1972):

(1) $$\begin{array}{c} \text{LDL}‐\text{C}=\text{TC}-\left(\frac{\text{Triglycerides}}{\left(5+\text{HDL}‐\text{C}\right)}\right). \end{array}$$

## 3. Results and Discussion

### 3.1. Antihyperglycemic Activities of Pumpkin Peel, Flesh, and Seed Powders

Table 2 shows data on the effects of pumpkin powders from 5, 10, and 15 g, respectively, following 28 days of intervention upon the blood glucose level of rats. Blood glucose level of different rats in 28 days of controlled study was checked at 7 days interval, and all three types of pumpkin powders (peel, flesh, and seeds) exhibited a significant decrease in blood glucose level of diabetic rats. Similarly, as the level of pumpkin powders was increased from 5 g to 10 g and 15 g, decrease in blood glucose level was found more convincing. From Table 2, it can be observed that in group A (normal rats with normal diet), mean value of bold glucose in 28 days was found 90.87 ± 0.65 mg/dl, and this blood glucose level was significantly increased in group B (diabetic rats fed with normal diet), and mean value of 28 days study was found 297.00 ± 0.95 mg/dl.

Application of 5 g, 10 g, and 15 g pumpkin peel powder caused nonsignificant decrease in blood glucose levels at the starting day of study as values were found 292.67 ± 5.46 mg/dl, 291.67 ± 6.01 mg/dl, and 294.33 ± 2.33 mg/dl, respectively; after 7 days of application of pumpkin peel powder, a significant decrease in blood glucose level was observed, whereas after 28 days of application of pumpkin peel powder, most significant decrease in blood glucose level of alloxan-induced diabetic rats was found as values were found 166.00 ± 2.52 mg/dl, 160.67 ± 1.76 mg/dl, and 147.67 ± 1.45 mg/dl for C, D, and E groups, respectively (Table 2).

Results of pumpkin flesh powder were also comparable with pumpkin peel powder with significant decrease in blood glucose level of alloxan-induced diabetic rats. Application of 5 g, 10 g, and 15 g pumpkin flesh powder caused nonsignificant decrease in blood glucose levels at the starting day of study as values were found 290.67 ± 2.33 mg/dl, 291.33 ± 2.03 mg/dl, and 291.33 ± 4.48 mg/dl for F, G, and H groups, respectively; after 7 days of application of pumpkin flesh powder, a significant decrease in blood glucose level was observed as values were found 287.67 ± 1.45 mg/dl, 282.33 ± 1.45 mg/dl, and 273.33 ± 1.67 mg/dl for F, G, and H groups, respectively, whereas after 28 days of application of pumpkin flesh powder, most significant decrease in blood glucose level of alloxan-induced diabetic rats was observed as values were found 172.33 ± 2.33 mg/dl, 157.67 ± 3.18 mg/dl, and 141.67 ± 1.67 mg/dl for F, G, and H group, respectively (Table 2).

More significant results were obtained in case of application of pumpkin seed powder as compared to pumpkin peel and flesh powder as decreasing effect towards blood glucose level in alloxan-induced diabetic rats was more prominent as after 28 days of application of pumpkin seed powder, most significant decrease in blood glucose level of alloxan-induced diabetic rats was observed as values were found 147.67 ± 3.76 mg/dl, 137.67 ± 1.45 mg/dl, and 128.33 ± 1.67 mg/dl for I, J, and K group, respectively (Table 2).

Antidiabetic components of different parts of pumpkin were listed by Adams et al. [41], and active components in lowering blood glucose level were pectin and nonpectin polysaccharides from peel and pulp of pumpkin and hypoglycemic proteins and seed oils from seeds of pumpkin. Protein bound polysaccharides have been reported to lower blood glucose level, increase plasma insulin level, and increasing glucose tolerance in alloxan-induced diabetic rats, and it is thought that this blood glucose-lowering effect of pumpkin polysaccharides might be due to antioxidant nature of polysaccharides which protects *β* cells of pancreas [42]. Pectin, which is present in pumpkin when is consumed, controls glycemic levels and reduces the need for insulin in patients with diabetes [43]. The high-glucose retardation effect of dietary fibers might be due to the high viscosity of soluble fraction which results in lesser glucose absorption by trapping glucose molecules in gel matrix developed by fibers; also, physical trapping of glucose molecules by fiber particles might influence retardation in glucose absorption [44, 45]. Besides pumpkin polysaccharides, inositol, zinc, chromium, cobalt, oil from ungerminated pumpkin seeds, and proteins from germinated pumpkin seeds possess hypoglycemic effect [46]. Trigonelline and nicotinic acids present in pumpkins also possess antidiabetic properties [47].

Our study results were supported by XueMin and Jue [48] as they carried out experiment on alloxan-induced diabetic rats by giving three types of pumpkin ingredients named as polysaccharide A, polysaccharide B, and nonpolysaccharide and compared their blood glucose level-lowering effect with Chinese standard drug Xiaoke. Another study on hypoglycemic effect of pumpkin (*Cucurbita maxima*) pulp powder, conducted by Mahmoodpoor et al. [49], helped to justify our results on blood glucose-lowering effect of pumpkin. They examined the effect of pumpkin pulp powder application besides insulin on control of blood glucose level in diabetic patients admitted to intensive care unit. Jun et al. [20] proved that pumpkin peels exhibit antidiabetic effect, when they extracted pectic polysaccharides from pumpkin peel and studied their blood glucose retardation index.

Antidiabetic effect of pumpkin seeds was also proved by Makni et al. [26] by studying the effect of pumpkin seed and falx seed mixture powder on alloxan-induced diabetic rats and blood glucose level in diabetic group of rats fed with pumpkin seed and flax seed mixture was significantly decreased in comparison to the diabetic rat groups fed with normal diet. They also noticed the significant increase in plasma insulin level in diabetic rat groups fed with pumpkin seed and flax seed mixture. They stated that changes in insulin might have been brought changes in hepatic glycogen content and lead to regulatory effect of flax and pumpkin seed mixture on glucose metabolism in alloxan-induced diabetic rats. Abuelgassim and Al-Showayman [50] studied the effect of pumpkin seed supplementation in diet on serum glucose level in atherogenic rats and found nonsignificant data of blood glucose level in atherogenic rats and rats fed with pumpkin seeds diet. Results supporting our study were present when Sayahi and Shirali [51] studied the effect of pumpkin (*Cucurbita pepo*) fruit extract on serum glucose concentrations and found a significant decrease in blood glucose level in diabetic groups treated with pumpkin fruit extract.

Another variety of *Cucurbitaceae* family (*Lagenaria siceraria*) known as white pumpkin was studied by Sharmin et al. [52], for its blood glucose-lowering effect in alloxan-induced diabetic rats, and this white pumpkin reduced fasting blood glucose level to 85.12, 58.82, and 34.60% at 4, 8, and 12 hours, respectively, after application of white pumpkin crude extract at a dose of 200 mg/kg body weight. Baldi et al. [53] studied the effect of pumpkin (*Cucurbita maxima*) concentrate on blood glucose level in alloxan-induced diabetic rats and observed a significant decrease in blood glucose level of rats. In normal rats, blood glucose level was 90.43 mg/dl; in alloxan-induced diabetic group of rats, blood glucose level was increased to 360.34 mg/dl, and it was reduced to 149.53 mg/dl when pumpkin concentrate was applied at 200 mg/kg body weight, and this blood glucose level was further decreased to 131.33 mg/dl when pumpkin concentrate was applied at 300 mg/kg body weight. Pumpkin concentrate enhances the activity of *β* cells of the pancreas which results in more insulin production, which causes decrease in blood glucose level.

#### 3.1.1. Antihyperlipidemic Studies of Pumpkin Peel, Flesh, and Seed Powders

Data for hypolipidemic study of different rats' groups fed with pumpkin peel, flesh, and seed powder at different levels has been presented in Table 3. Serum lipid composition after 28 days of controlled study was checked, and from Table 3, it was clear that all three types of pumpkin powders (peel, flesh, and seeds) caused a significant decrease in serum TC, TG, and LDL-C (bad cholesterols) and a significant increase in HDL-C (good cholesterol). Similarly, as the level of pumpkin powders was increased from 5 g to 10 g and 15 g, decrease in TC, TG, and LDL-C became more prominent, and increase in HDL-C was also elevated. From Table 3, it can be observed that in group A (normal rats with normal diet), TC was 80.59 ± 0.23 mg/dl, TG was 65.47 ± 0.20 mg/dl, HDL-C was 55.26 ± 0.22 mg/dl, and LDL-C was 20.69 ± 0.07 mg/dl, whereas in group B (diabetic rats with normal diet) TC, TG, and LDL-C were significantly increased as values were 103.28 ± 0.68 mg/dl, 85.78 ± 0.68 mg/dl, and 27.00 ± 0.08 mg/dl, respectively, and HDL-C level was significantly decreased to 52.38 ± 0.21 mg/dl.

Application of 5 g, 10 g, and 15 g pumpkin peel powder caused significant decrease in TC as values were found 97.31 ± 0.65 mg/dl, 95.21 ± 0.52 mg/dl, and 93.54 ± 1.03 mg/dl, respectively, significant decrease in TG as values were found 77.43 ± 0.56 mg/dl, 75.81 ± 0.99 mg/dl, and 73.58 ± 1.19 mg/dl, respectively, and significant decrease in LDL-C as values were found 24.17 ± 0.04 mg/dl, 23.53 ± 0.25 mg/dl, and 22.24 ± 0.16 mg/dl, respectively, whereas a significant increase in HDL-C was noticed as values were found 60.37 ± 0.55 mg/dl, 62.16 ± 0.56 mg/dl, and 64.63 ± 0.91 mg/dl, respectively (Table 3).

Results of pumpkin flesh powder were also comparable with pumpkin peel powder with significant decrease in bad cholesterols (TC, TG, and LDL-C) and significant increase in good cholesterol (HDL-C). Application of 5 g, 10 g, and 15 g pumpkin flesh powder caused significant decrease in TC as values were found 98.68 ± 0.52 mg/dl, 97.01 ± 0.64 mg/dl, and 94.53 ± 0.55 mg/dl, respectively, significant decrease in TG as values were found 79.36 ± 0.54 mg/dl, 77.52 ± 0.55 mg/dl, and 75.21 ± 1.02 mg/dl, respectively, and significant decrease in LDL-C as values were found 25.83 ± 0.12 mg/dl, 25.00 ± 0.05 mg/dl, and 23.85 ± 0.09 mg/dl, respectively, whereas a significant increase in HDL-C was noticed as values were found 59.22 ± 0.65 mg/dl, 61.65 ± 1.04 mg/dl, and 63.41 ± 0.57 mg/dl, respectively (Table 3).

**Figure 2:**
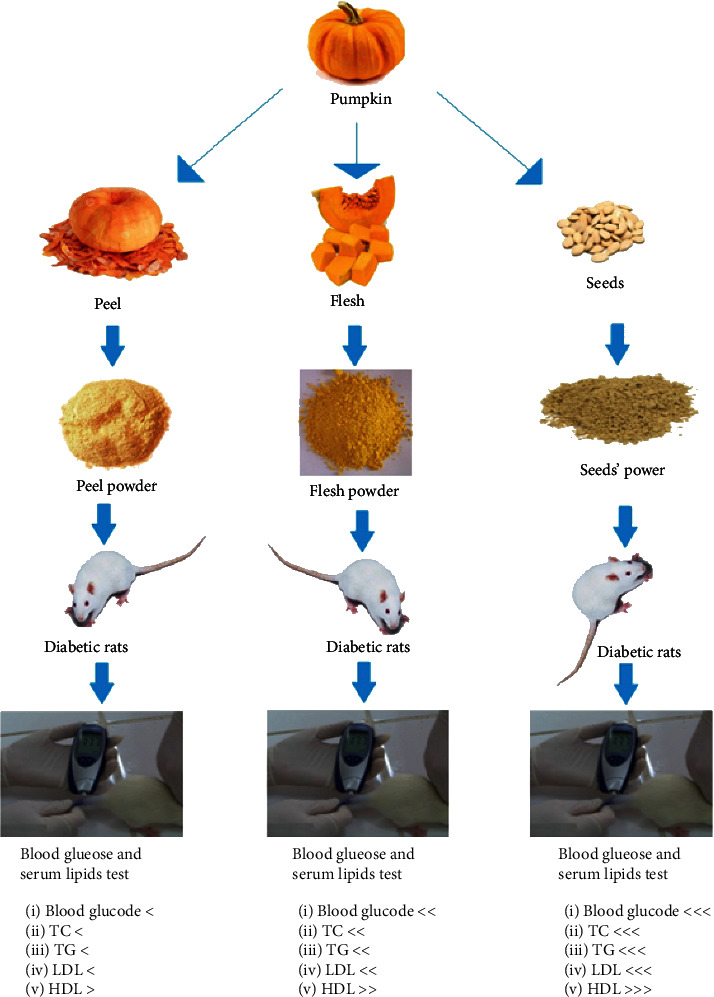
Graphical overview of research work.

More significant results were obtained in case of application of pumpkin seed powder as compared to pumpkin peel and flesh powder as decreasing effect towards bad cholesterols (TC, TG, and LDL-C), and increasing effect towards good cholesterols (HDL-C) was more prominent. Application of 5 g, 10 g, and 15 g pumpkin flesh powder caused significant decrease in TC as values were found 93.25 ± 0.39 mg/dl, 91.28 ± 0.68 mg/dl, and 88.43 ± 0.66 mg/dl, respectively, significant decrease in TG as values were found 73.32 ± 0.62 mg/dl, 71.45 ± 0.94 mg/dl, and 69.79 ± 0.49 mg/dl, respectively, and significant decrease in LDL-C as values were found 23.65 ± 0.09 mg/dl, 22.68 ± 0.09 mg/dl, and 21.45 ± 0.08 mg/dl, respectively, whereas a significant increase in HDL-C was noticed as values were found 63.33 ± 0.96 mg/dl, 65.18 ± 0.62 mg/dl, and 67.52 ± 0.55 mg/dl, respectively (Table 3). Graphical overview of research work has been presented in Figure 2.

Diabetes mellitus is one of the most common metabolic disorders, which also results in derangements in lipid metabolism, which are determinants of status and severity of the disease [54]. The hypolipidemic action of soluble dietary fiber fraction could be the result of the retardation of carbohydrate and fat absorption due to presence of bioactive fiber in the agent [55]. Polyunsaturated fatty acids such as oleic acid, linolenic acid, and linoleic acid present in pumpkins play a crucial role in lowering blood cholesterol level in humans and rats [56]. Hypoglycemic proteins present in pumpkin seeds and polysaccharides in pumpkin peel and pulp exhibit hypoglycemic activity in animals by lowering plasma lipid concentrations .

Ihedioha et al. [57] gave the optimal value of TC and TG 6.29 mmol/l and 4.22 mmol/l, respectively, in Wistar albino rats of comparable age and sex. LDL-C elevation in the blood is associated with several pathological conditions including diabetes, cardiovascular diseases, and inflammation, whereas HDL-C-elevated levels in the blood have been reported having positive effects on cardiovascular health [58].

Baldi et al. [53] studied the effect of pumpkin (*Cucurbita maxima*) concentrate on serum cholesterol level in alloxan-induced diabetic rats and observed a significant decrease in serum cholesterol level of rats. Another study conducted by Zhao et al. [59] on hypoglycemic effect of pumpkin polysaccharides extracted from pumpkins witnessed our results. Abuelgassim and Al-Showayman [50] studied the effect of pumpkin seed supplementation in diet on serum lipid levels in atherogenic rats and found a significant decrease in TC and LDL-C. TC in atherogenic rats was 4.89 mmol/l, and it was decreased 48% (2.55 mmol/l) in rats treated with pumpkin seeds, whereas LDL-C in atherogenic rats was 3.33 mmol/l and in pumpkin seeds fed diet rat groups, it was reduced to 0.70 mmol/l which becomes 79% decrease. Nonsignificant data was found for TG but a significant increase in HDL-C was observed as in atherogenic rats, HDL-C was 0.43 mmol/l, and it was increased to 0.89 mmol/l. Hypolipidemic effect of pumpkin seeds was also proved by Makni et al. [26] by studying the effect of pumpkin seed and falx seed mixture powder on alloxan-induced diabetic rats and found an increase in plasma and liver lipids in diabetic group of rats fed with normal diet, by 108% and 30%, respectively, but in rat groups fed with pumpkin and flax seed mixture, both plasma and liver lipids were decreased by 21% and 19% as compared to diabetic group of rats fed with normal diet.

Mohamed et al. [60] studied the effect of substituting pumpkin seed protein isolate at 25, 50, 75, and 100% level with casein on serum lipid levels in CCl_4_-intoxicated rats and found supporting results. Sayahi and Shirali [51] studied the effect of pumpkin (*Cucurbita pepo*) fruit extract on serum lipid concentrations, and contradictory results for TG and TC were obtained with a nonsignificant increase in TG as in diabetic group, TG were 92.40 mg/dl, and in group treated with pumpkin extract, TG were 93.16 mg/dl, and a significant increase in TC as in diabetic group TC was 66.60 mg/dl, and it was increased to 73.83 mg/dl in diabetic group treated with pumpkin fruit extract. Results for LDL-C and HDL-C were similar to our study as an increase in HDL-C was observed (diabetic rats' group: 34.28 mg/dl and pumpkin extract-treated group: 38.76 mg/dl), and decrease in LDL-C was observed (diabetic rats' group: 14.60 mg/dl and pumpkin extract-treated group: 13.16 mg/dl).

Another variety of Cucurbitaceae family (*Lagenaria siceraria*) known as white pumpkin was studied by Sharmin et al. [52] for its serum lipid-lowering effect in alloxan-induced diabetic rats, and this white pumpkin reduced elevated TC level in blood to 14.91%, elevated serum TG level to 68.24%, and elevated serum LDL-C level to 27.67%. Eneh et al. [61] studied the effect of ethanol extract of pumpkin (*Cucurbita pepo*) leaves on lipid concentrations in Wistar albino rats. TC (1.06 mmol/l) and TG (0.782 mmol/l) were significantly increased in test group (rats fed with 10% pumpkins leave concentrate) as compared to baseline group (rats sacrificed just after purchasing) in which TC and TG were 0.28 mmol/l and 0.326 mmol/l, respectively. This increase in TC and TG might be suggested due to lean-diet fed to the rats from where these were purchased. They observed a significant decrease in LDL-C which was 0.11 mmol/l in baseline group and was decreased to 0.05 mmol/l in test group and a significant increase in HDL-C which was 0.30 mmol/l in baseline group and was increased to 0.50 mmol/l in test group.

## 4. Conclusion

Pumpkin constituent parts are excellent sources of bioactives. Pumpkin peel, flesh, and seeds are rich source of phytochemicals, which are capable of playing medicinal role in animals and humans. Pumpkin part powder incorporation in diet of alloxan-induced diabetic animals reduced the blood glucose and lipid levels significantly. Antihyperglycemic and antihyperlipidemic activities of pumpkin powders are proof of this reality that no part of pumpkin fruit can be considered as waste material in order to promote healthy eating; food products developed by incorporation of pumpkin could be a key strategy to develop a healthy community.

## Figures and Tables

**Table 1:** Treatment plan for biological study of pumpkin peel, flesh, and seed powders.

Ingredients	Negative control Normal rats	Positive control Diabetic rats	Positive control Diabetic rats fed with peel powder			Positive control Diabetic rats fed with flesh powder			Positive control Diabetic rats fed with seed powder		
	A	B	C	D	E	F	G	H	I	J	K
Pumpkin peel powder (g)	0	0	5	10	15	0	0	0	0	0	0
Pumpkin flesh powder (g)	0	0	0	0	0	5	10	15	0	0	0
Pumpkin seed powder (g)	0	0	0	0	0	0	0	0	5	10	15
Casein (g/100 g)	26	26	26	26	26	26	26	26	26	26	26
Corn starch (g/100 g)	50	50	45	40	35	45	40	35	45	40	35
Sucrose (g/100 g)	9	9	9	9	9	9	9	9	9	9	9
Cellulose (g/100 g)	5	5	5	5	5	5	5	5	5	5	5
Vitamin mix (g/100 g)	1	1	1	1	1	1	1	1	1	1	1
Mineral mix (g/100 g)	4	4	4	4	4	4	4	4	4	4	4
Corn oil (g/100 g)	5	5	5	5	5	5	5	5	5	5	5

**Table 2:** Antihyperglycemic activity parameters of pumpkin peel, flesh, and seed powders.

Group^∗^	Means (±SE) of blood glucose level (mg/dl)				
	0 day	7 days	14 days	21 days	28 days
A	90.00 ± 1.15y	90.67 ± 0.67y	92.67 ± 1.45y	91.00 ± 0.58y	90.00 ± 2.89y
B	296.00 ± 3.06ab	294.33 ± 2.33abc	297.67 ± 1.45ab	299.33 ± 2.33a	297.67 ± 1.45ab
C	292.67 ± 5.46abc	284.33 ± 2.33de	205.00 ± 2.89j	172.33 ± 1.45op	166.00 ± 2.52pq
D	291.67 ± 6.01bcd	274.33 ± 2.33f	195.00 ± 2.89k	166.00 ± 2.08pq	160.67 ± 1.76qr
E	294.33 ± 2.33abc	271.67 ± 1.67f	184.33 ± 2.33lmn	153.00 ± 1.73st	147.67 ± 1.45tu
F	290.67 ± 2.33bcd	287.67 ± 1.45cde	212.00 ± 1.53j	179.33 ± 2.33no	172.33 ± 2.33op
G	291.33 ± 2.03bcd	282.33 ± 1.45e	189.67 ± 2.91kl	157.33 ± 3.71rs	157.67 ± 3.18rs
H	291.33 ± 4.48bcd	273.33 ± 1.67f	181.67 ± 1.20mn	145.67 ± 3.48tu	141.67 ± 1.67uv
I	293.00 ± 5.69abc	263.33 ± 1.67g	195.00 ± 2.89k	152.00 ± 1.53st	147.67 ± 3.76tu
J	293.67 ± 3.48abc	255.33 ± 2.91h	187.67 ± 2.60klm	137.67 ± 3.93vw	137.67 ± 1.45vw
K	295.67 ± 2.96ab	246.67 ± 1.67i	179.33 ± 1.76no	132.33 ± 1.45wx	128.33 ± 1.67x
Overall means	274.58 ± 10.37A	256.73 ± 9.59B	192.73 ± 7.92C	162.36 ± 8.66D	158.85 ± 8.64E

Means sharing same letter in a column are statistically nonsignificant, and means sharing different letters in a column are statistically significant (*P* > 0.05). Small letters represent comparison among interaction means, and capital letters are used for overall mean. Group^∗^; A: negative control (normal rats with normal diet), B: positive control (diabetic rats with normal diet), C: rat groups fed with 5 g pumpkin peel powder, D: rat groups fed with 10 g pumpkin peel powder, E: rat groups fed with 15 g pumpkin peel powder, F: rat groups fed with 5 g pumpkin flesh powder, G: rat groups fed with 10 g pumpkin flesh powder, H: rat groups fed with 15 g pumpkin flesh powder, I: rat groups fed with 5 g pumpkin seed powder, J: rat groups fed with 10 g pumpkin seed powder, K: rat groups fed with 15 g pumpkin seed powder.

**Table 3:** Antihyperlipidemic activity parameters of pumpkin peel, flesh, and seed powders.

Groups^∗^	Means (±SE) of lipids			
	TC (mg/dl)	TG (mg/dl)	HDL-C (mg/dl)	LDL-C (mg/dl)
A	80.59 ± 0.23h	65.47 ± 0.20h	55.26 ± 0.22f	20.69 ± 0.07i
B	103.28 ± 0.68a	85.78 ± 0.68a	52.38 ± 0.21g	27.00 ± 0.08a
C	97.31 ± 0.65b	77.43 ± 0.56bcd	60.37 ± 0.55de	24.17 ± 0.04d
D	95.21 ± 0.52cd	75.81 ± 0.99cd	62.16 ± 0.56cd	23.53 ± 0.25e
E	93.54 ± 1.03de	73.58 ± 1.19ef	64.63 ± 0.91b	22.24 ± 0.16g
F	98.68 ± 0.52b	79.36 ± 0.54b	59.22 ± 0.65e	25.83 ± 0.12b
G	97.01 ± 0.64bc	77.52 ± 0.55bc	61.65 ± 1.04cd	25.00 ± 0.05c
H	94.53 ± 0.55de	75.21 ± 1.02de	63.41 ± 0.57bc	23.85 ± 0.09de
I	93.25 ± 0.39e	73.32 ± 0.62ef	63.33 ± 0.96bc	23.65 ± 0.09e
J	91.28 ± 0.68f	71.45 ± 0.94fg	65.18 ± 0.62b	22.68 ± 0.09f
K	88.43 ± 0.66g	69.79 ± 0.49g	67.52 ± 0.55a	21.45 ± 0.08h

Means sharing same letter in a column are statistically nonsignificant, and means sharing different letters in a column are statistically significant (*P* > 0.05). Groups^∗^; A: negative control (normal rats with normal diet), B: positive control (diabetic rats with normal diet), C: rat groups fed with 5 g pumpkin peel powder, D: rat groups fed with 10 g pumpkin peel powder, E: rat groups fed with 15 g pumpkin peel powder, F: rat groups fed with 5 g pumpkin flesh powder, G: rat groups fed with 10 g pumpkin flesh powder, H: rat groups fed with 15 g pumpkin flesh powder, I: rat groups fed with 5 g pumpkin seed powder, J: rat groups fed with 10 g pumpkin seed powder, K: rat groups fed with 15 g pumpkin seed powder.

## Data Availability

Data available on request.

## Additional Points

*Practical Applications*. Peel, flesh, and seeds of pumpkins are well equipped with nutraceuticals and functional ingredients. These fractions of pumpkin, instead of discarding, must be utilized as pharma foods. Pumpkin parts are capable of exhibiting antihyperlipidemic, antihyperglycemic, immunomodulatory, antihypertensive, antimicrobial, and antioxidant potential in humans. Need of healthy eating in current post COVID 19 period is crucial and medicinal foods like pumpkin, and bioactive compounds present in this functional food could play a vital role in developing healthy community. Development of food products by incorporation of pumpkin could generate a new variety of sustainable, health-promoting food stuffs. Phytochemicals from pumpkin fractions could be extracted, and controlled clinical trials on humans with different ailments can be carried out to evaluate the potential therapeutic and prophylactic efficacy of the extracts.
